# Panel and Panelist Performance in the Sensory Evaluation of Black Ripe Olives from Spanish Manzanilla and Hojiblanca Cultivars

**DOI:** 10.3390/foods8110562

**Published:** 2019-11-08

**Authors:** Antonio López-López, Antonio Higinio Sánchez-Gómez, Alfredo Montaño, Amparo Cortés-Delgado, Antonio Garrido-Fernández

**Affiliations:** Food Biotechnology Department, Instituto de la Grasa (CSIC), Campus Universitario Pablo de Olavide, Edificio 46, Ctra. Utrera km 1, 41013 Sevilla, Spain; ahiginio@ig.csic.es (A.H.S.-G.); amontano@cica.es (A.M.); acortes@cica.es (A.C.-D.); garfer@cica.es (A.G.-F.)

**Keywords:** panel performance, panelist, black ripe table olives, sensory descriptors, sensory profile

## Abstract

There is vast experience in the application of sensory analysis to green Spanish-style olives, but ripe black olives (≈1 × 10^6^ kg for 2016/2017) have received scarce attention and panelists have less experience on the evaluation of this presentation. Therefore, the study of their performance during the assessment of this presentation is critical. Using previously developed lexicon, ripe olives from Manzanilla and Hojiblanca cultivars from different origins were sensory analysed according to the Quantitative Descriptive Analysis (QDA). The panel (eight men and six women) was trained, and the QDA tests were performed following similar recommendations than for green olives. The data were examined while using SensoMineR v.1.07, programmed in R, which provides a diversity of easy to interpret graphical outputs. The repeatability and reproducibility of panel and panelists were good for product characterisation. However, the panel performance investigation was essential in detecting details of panel work (detection of panelists with low discriminant power, those that have interpreted the scale in a different way than the whole panel, the identification of panelists who required training in several/specific descriptors, or those with low discriminant power). Besides, the study identified the descriptors of hard evaluation (skin green, vinegar, bitterness, or natural fruity/floral).

## 1. Introduction

World table olive production was around 2.6 × 10^6^ tones in season 2016/2017 according to the last consolidated balance of the International Olive Oil Council [1]. Approximately, 40% of them were processed as black ripe table olives (Californian style). This style was first developed in the USA, which is still one of the most relevant contributors with current production of about 80 × 10^3^ tons [1], but other countries, like Spain, Greece, Turkey, or Egypt, are progressively increasing their productions. Black ripe table olive processing includes a phase of storage, which is usually accomplished by immersing the fruits in brine or acidified solution, followed by a darkening step, which consists of the application of one (or several) lye treatments and subsequent immersion in tap water to remove the excess of alkali. During this oxidation phase, air is also bubbled through the suspension to accelerate browning. The colour is then fixed by a ferrous gluconate solution, after which the olives are packed and the cans sterilised [2]. The products usually offer a rather plain organoleptic profile, which has been a favourable condition for its introduction in new markets, due to their numerous treatments in aqueous solutions. In fact, according to the Trade Standards Applying to Table Olives [3], the only requisites for these olives are sensory characteristics and texture in agreement with their processing system.

Along the last decade, the International Olive Council developed a method for the sensory evaluation of table olives. However, it was mainly focused on green Spanish-style, since most of the descriptors included in the evaluation sheet are exclusively related to this product (e.g., abnormal fermentation, acidity, or bitterness) [4]. However, methods for the evaluation and classification of black ripe olives were developed in California, where this processing has a long tradition [5].

On the other hand, Quantitative Descriptive Analysis (QDA) is widely used for studying the sensory profile of diverse foods ([6,7,8], among many others). Recently, researchers have applied QDA to a list of 33 descriptors for the sensory comparison of American black ripe table olives with respect to those that are imported from other countries (Spain, Egypt, or Morocco) [9]. Similar descriptors were used to study the sensory profile of black ripe table olives from Spanish Manzanilla and Hojiblanca cultivars and successfully distinguishing among cultivars, farming origins, and storage period [10]. López-López et al. [11] have developed an entirely new lexicon for the application of QDA to Spanish-style green table olives; the results showed relevant differences between cultivars and origins. Therefore, the use of the QDA to black ripe table olives from the most important Spanish cultivar devoted to this elaboration is relevant.

Traditionally, the sensory analysis of table olive, regardless of style, has been mainly devoted to the characterization of products [12,13,14,15,16], but the panelists and panel performances were rarely studied in detail. However, along the last two decades, different authors have developed methodologies for evaluating the reliability of the panel [17,18,19,20,21,22]. Its application to the panel performance, discrimination power of descriptors of diverse green and black ripe table olives, following the COI/OT/MO No. 1/Rev. 2 methodology, has been recently published [17]. Nevertheless, the performance of a panel and panelists that were devoted to the sensory analysis of black ripe table olives using QDA has never been studied.

This work aims for the application of Quantitative Descriptive Analysis to black ripe table olives from Spanish Manzanilla and Hojiblanca cultivars, focusing interest on the panel and panelist performances as a tool for improving their training and reliability.

## 2. Materials and Methods 

### 2.1. Olives and Their Processing

The olives were of the Manzanilla and Hojiblanca cultivars, harvested at green maturation stage in October 2016. Their origins were: Aljarafe (Sevilla) and Lora de Estepa (Sevilla) for *Manzanilla*, and Lora de Estepa (Sevilla), and Alameda (Málaga) for *Hojiblanca*. The samples were identified as MAL, ML, HL, and HA, according to cultivar (initial letter) and growing area (remaining letter/s).

Just harvested olives from each cultivar and origin were directly brined in 25 L (15 kg olives) PVC (polyvinyl chloride) fermenters in an acidified (2.4% acetic acid) solution. After three months of storage, the fruits were subjected to the darkening process. For this purpose, horizontal stainless steel cylindrical containers (0.4 m diameter, 0.7 m length) were used. The fruits were treated with a 3% lye solution until the alkali reached the pit. After removing the alkali, the olives were washed to low the pH up to 8.0 units. During both operations, an oxygen-saturated ambient was maintained in the suspension by bubbling air through a perforated tube lying along the bottom of the oxidation vessels. Subsequently, the black colour developed was fixed, while using a 0.1% ferrous gluconate solution with pH adjusted to 4.5 to prevent the precipitation of the element as hydroxide. Afterwards, the darkened olives were introduced in glass jars (145 g of olives), together with 170 mL of 3.5% NaCl cover solution, which also contained 0.2 g ferrous gluconate/L and had the pH adjusted to 4.5 with acetic acid. Finally, the jars were closed and sterilised at 130 °C for 20 min [23].

The sensory analysis of the above-prepared black ripe olives was achieved after storage at room temperature for 30 (to allow complete olive flesh/brine equilibrium) and 210 days (estimated maximum normal period of the product in the shelves before reposition). The new codes were those previously mentioned, plus 1 (one-month storage) and 2 (seven-month storage), respectively. Therefore, the symbols of the final samples: were: MAL1, MAL2, ML1, ML2, HL1, HL2, HA1, and HA2, which indicated the successive letters and figures cultivar, growing area, and the storage period, respectively.

A panel composed of eight men and six women, making a total of 14 panelists (40 years’ average age) performed the analysis. They all belonged to the Instituto de la Grasa staff and had vast experience on sensory studies due to their participation in the development of the Sensory Analysis Method for Table Olives [4] and the permanent involvement in diverse IG table olive sensory projects (e.g., [10,11]). Before the tests, the panelists were trained for one h twice a week for two months to familiarise them with the QDA techniques and the black ripe olive descriptors, while using industrially processed Spanish cultivars black ripe olives. The presentation of the samples was always made in the standard glasses [24], which were coded with three randomly chosen digits. After each test, the mouth was washed with tap water, freely available in each booth. Therefore, the panelists were progressively familiarised with the product, the sensory descriptors that were included in the evaluation sheet, informal tentative evaluations, and, finally, allowed for practicioning with the unstructured scale (1, complete absence; 11, strongest perception) of the evaluation sheet for another month. After these periods, they were considered ready for the evaluation of the real samples because of the previous expertise of the panelists in sensory testing. The assessed descriptors included appearance (skin red, skin green, skin sheen, flesh red, flesh yellow, and flesh green), aroma (briny, mushroom, earth/soil, oak/barrel, nutty, artificial fruity/floral, natural fruity/floral, vinegary, alcohol, fishy smell/ocean, and cheese smell), taste (sourness, bitterness, and saltiness), flavor (ripeness, buttery, metallic, rancid, soapy smell/medicinal, and gassy smell), and texture/mouthfeel (firmness, fibrousness, moisture release, mouth coating, chewiness, astringency, and residual). Their definitions and references may be found elsewhere [10].

For performing the tests, the black ripe olive samples were presented to panelists at an ambient temperature (20 ± 1 °C) and in a panel room that was equipped with individual booths under incandescent white lighting and free from any odors. The panelists were asked to mark the intensity of the different descriptors in the evaluation sheets. The scores of the attributes were measured with the exactitude of one decimal point and the results tabulated.

### 2.2. Data Analysis

The data were mainly studied while using the SensoMineR v.1.07 software (Agrocampus Ouest, Rennes, France) [25], a package that was designed and programmed in R language [26]. It is characterized by combining classical sensory statistical methods as well as others directly conceived in the developers’ laboratory. In this way, SensoMineR provides a synthesis of the results of the usual analysis of variance (ANOVA) models, as well as a diversity of easy to interpret graphical outputs. Notably, the package includes several options for the panel evaluation, such as multivariate analysis and the generation of virtual panels, by bootstrapping techniques, which allow for the estimation of the corresponding confidence limits. XLSTAT [27] was also applied in specific analysis and tests.

## 3. Results and Discussion

The matrix of data was constituted by the following variables: sample-storage period (just sample from now on), panelist, session, and the 33 descriptors making a total of 36 columns. Additionally, sample, panelist, and session had 8, 14, and 3 levels, respectively, making a total of 336 rows. Therefore, the overall number of cells was 12,096. The generated database was already used for product characterization [10], but, in this work, the analysis is focused on the panel and panelists performance as an exercise for improving their evaluation and training.

### 3.1. Overview of Results

After checking the dataset for possible outliers and typing errors, they were also subjected to a first overview (frequency histograms and boxplots), which indicates that several descriptors received low scores and they were hardly noticed; however, others were perceived by the panelists, distributed along the scale, and allowed for discrimination among samples (data not shown). Further details can be found elsewhere [10].

### 3.2. Panel Performance

The techniques that are available for panel and panelists performance are numerous, with ANOVA and multivariate analysis being the most common. Kermit and Lengard Almli [16] presented univariate and multivariate data analysis methods to assess the individual and group performances in a sensory panel. Notably, Husson et al., [25] developed the SensoMineR, which includes several innovative tools with this objective.

#### 3.2.1. Effect of Sample (Power of Discrimination)

The evaluation of the panel performance is an essential premise not only for obtaining reliable results on sensory analysis, but also for improving the selection of panelists and their training. In this work, the *panelperf* instruction from SensoMineR, with the appropriate models and the corresponding analysis of variance, was used. The ANOVA was fitted to the following full model:

Score = sample + panelist + session + sample panelist + sample session + panelist session

where score stands for the expected evaluation value, while sample, panelist, and session for the predictive variables, with the effect of storage being included as levels of the variable sample. The panelist and the session were both studied as random effects, but the sample was considered to be fixed [28].

The results regarding performance (Table 1) showed that the panel was able to discriminate the samples based on skin green, flesh green, skin sheen, flesh red, firmness, fibrousness, flesh yellow, skin red, vinegary, moisture release, fishy smell/ocean, and saltiness. Good segregation among the samples or products by panelists is systematically reported in numerous publications ([6,17,28,29,30], among others).

#### 3.2.2. Effect of Panelist

The significant effect of the panelist, with very low *p*-values, regardless of descriptors, indicates a different interpretation of the scales. Such an effect is not desirable, but it is usually observed. However, its presence does not represent any inconvenience for achieving appropriate conclusions, since the panelists’ variance can be eliminated thanks to the ANOVA analysis and by centring the data with respect to panelists [31]. The assessors’ performance will be studied in detail later.

#### 3.2.3. Effect of Session

The effect of the session was not significant for any descriptor (Table 1), which indicates an overall good panelist performance over time (the samples were assessed in the same way from one session to another), which is an appropriated and desired situation. Subsequently, no further comments regarding this aspect are also required.

#### 3.2.4. Sample·Panelist Interaction

**Figure 1:**
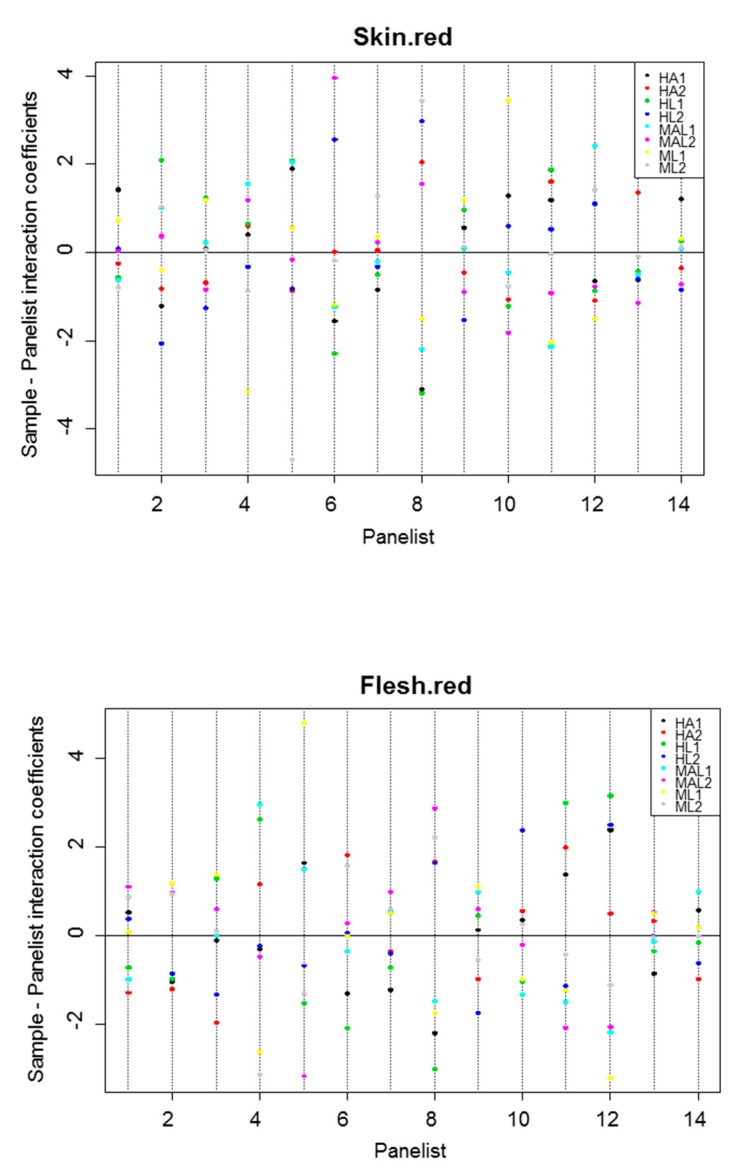
Panel performance. Sample·panelist interaction coefficients for selected descriptors (skin red and flesh red).

In the case of a total consensus among the members of the panel to assess the descriptors in all samples, their effects should not be significant. However, in this work, there were numerous significant cases (Table 1). The evaluation of the interaction is usually measured by the coefficients of the ANOVA, defined as the difference between the expected mean score by all panelists and that given by a specific one. It is tedious to reproduce their meaning in all descriptors, so only the case of skin red and flesh red are shown as examples (Figure 1). The effect might be significant because of two circumstances: (i) the panelists do no rank the samples in the same order and (ii) they do no use the scale in the same way. Both situations were found in this work. Examples of different ranks were observed, among other descriptors, for skin red, panelist 1 gave the highest score to HA1, but panelist 2 ranked it as the second one from the bottom; a similar behaviour occurred for flesh red regarding panelist 5 with respect to panelist 6 (Figure 1).

On the other side, for skin red, panelist 1 used a narrower scale than panelist 6; the same trend can be observed for flesh red by panelist 1 and panelist 12 (Figure 1). Therefore, to improve panel performance, it will be required further additional training in the scoring of some attributes and the amplitude of their scales.

**Figure 2:**
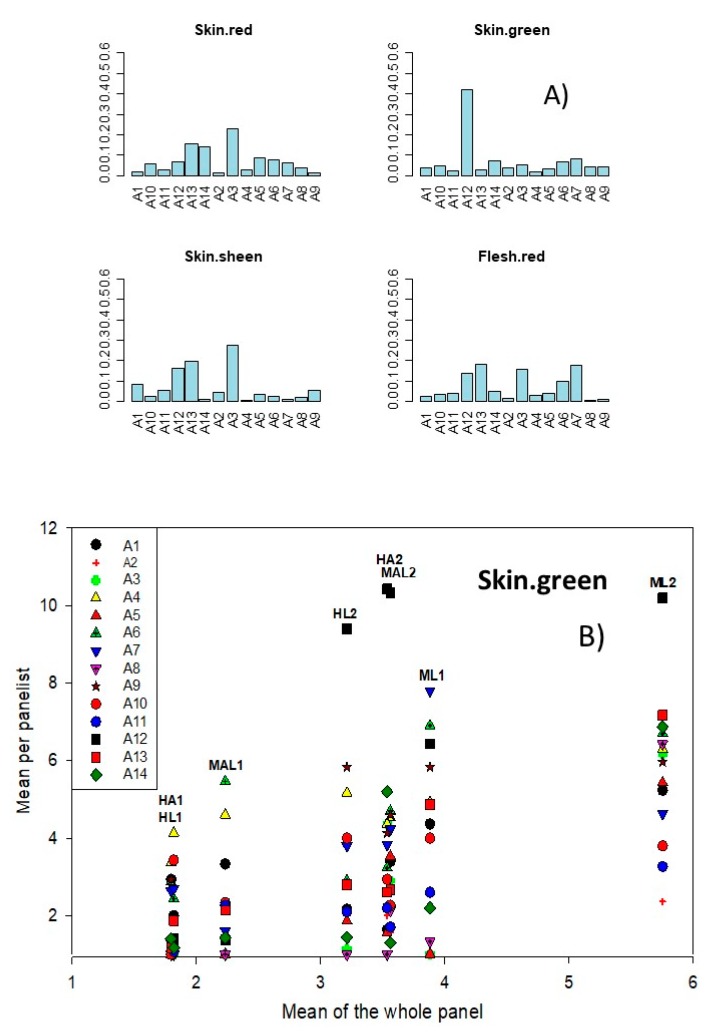
Panel performance. Sample·panelist interaction as assessed by (**A**) the panelist’s contributions (coefficients) for selected descriptors (skin red, skin green, skin sheen, and flesh red), and (**B**) means of panelists over the whole panel according to samples.

The corresponding coefficients of each panelist in the ANOVA model were assessed by the identification of the panelists who mainly contributed to the interaction [19]. With this aim, the difference between the expected score and that given by a concrete panelist, overall sessions and samples, represent how far a specific panelist scores the sample differently to the product mean of the whole panel. No significant differences were usually observed (panelists had, in general, good reproducibility), but some peculiarities were noticed. For example, panelist A12 scored skin green (Figure 2A) sensibly higher than any other panelist; subsequently, he was critical in the significance of this interaction. Additionally, panelist A3 tends to scoring skin red, skin sheen, and flesh red above the panel average (Figure 2A).

Another way of observing the sample·panelist interaction and measuring the panelists’ reproducibility is by plotting the mean per panelist over the mean on the whole panel according to samples. In agreement with previous comments, some panelists gave high scores to several descriptors and, in this line, panelist A12 overscored skin green in samples HL2, HA2, MAL2, and ML2 (Figure 2B). These high scores were due to a tendency of this panelist to evaluate several descriptors (flesh yellow and briny, data not shown) higher than other panel members. Similarly, outstanding scores were observed for panelist A5 in vinegary, alcohol, and sourness, and for panelist A8 in mouth coating, chewiness, stringency, and residual (data not are shown). However, most of the panelists differently scored only one descriptor like A4 in grassy smell, A10 in cheesy smell, A3 in a buttery, or A6 in rancid, to mention a few cases. Therefore, no panelist systematically contributed to the interaction, but the above-mentioned results could indicate that the panel performance would be improved by the further training of some panel members (A12, A5, and A8, on several descriptors or A4, A10, A3, or A6, only regarding specific ones). Kermit and Lengard Almli [19] also found several assessors who showed poor performance in some attributes, such as mealiness or fruity flavor.

#### 3.2.5. Sample·Session Interaction

**Figure 3:**
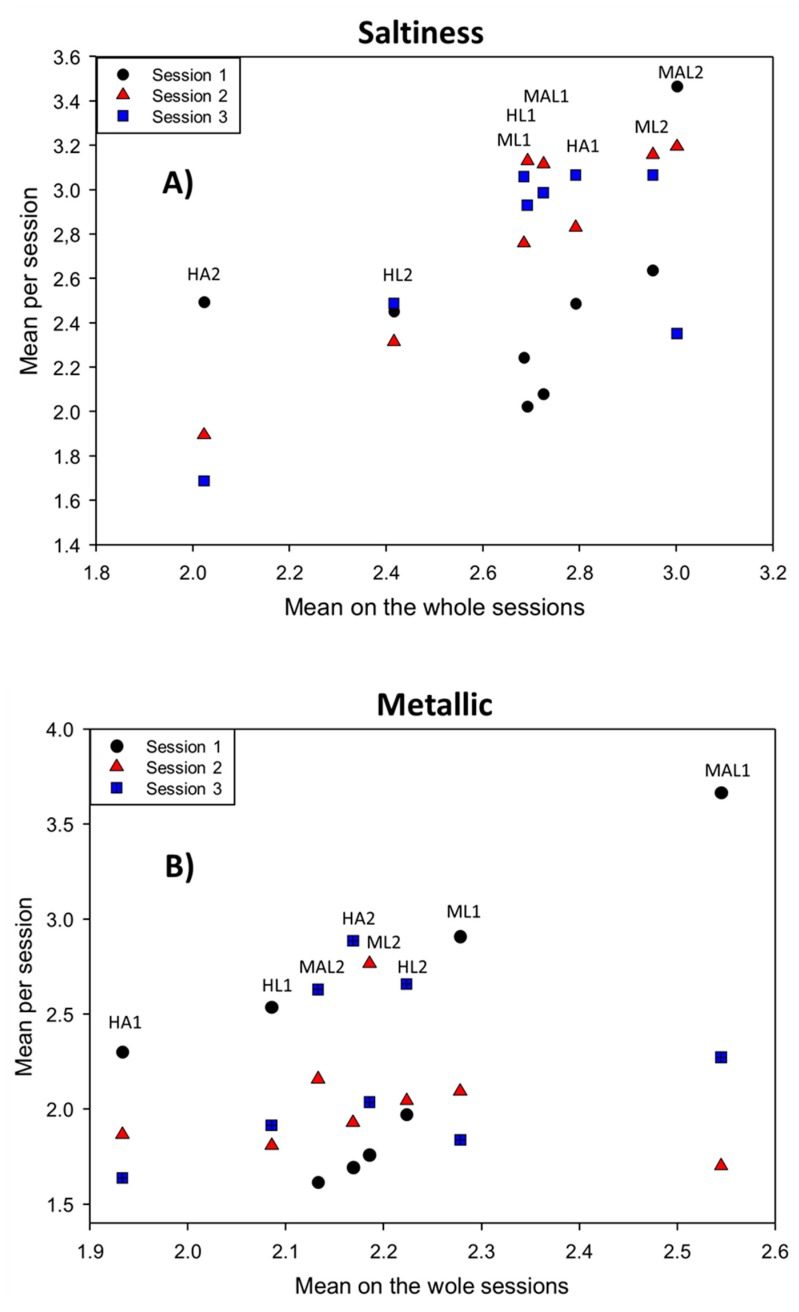
Panel performance. Sample·session interaction. Mean per session of panelists, according to samples, over the sample means of the whole sessions for significant descriptors: (**A**) saltiness, and (**B**) metallic.

These interactions refer to the variation of the mean of each sample from one session to another and they should not be confused with the session effect, which applies to the mean of all samples between sessions. In the study (Table 1), the sample·session interaction was only significant in two cases: saltines (which was an important descriptor for sample discrimination) and metallic (Table 1). In saltiness, the significant interaction was mainly produced because of the different scoring for samples HA2, HL1, HA1, MAL1, and MAL2 in session S1 (Figure 3), while, in the case of metallic, the significant interaction is due to the abnormally high score of MAL1 in session S1 (Figure 3).

#### 3.2.6. Panelist·Session Interaction

**Figure 4:**
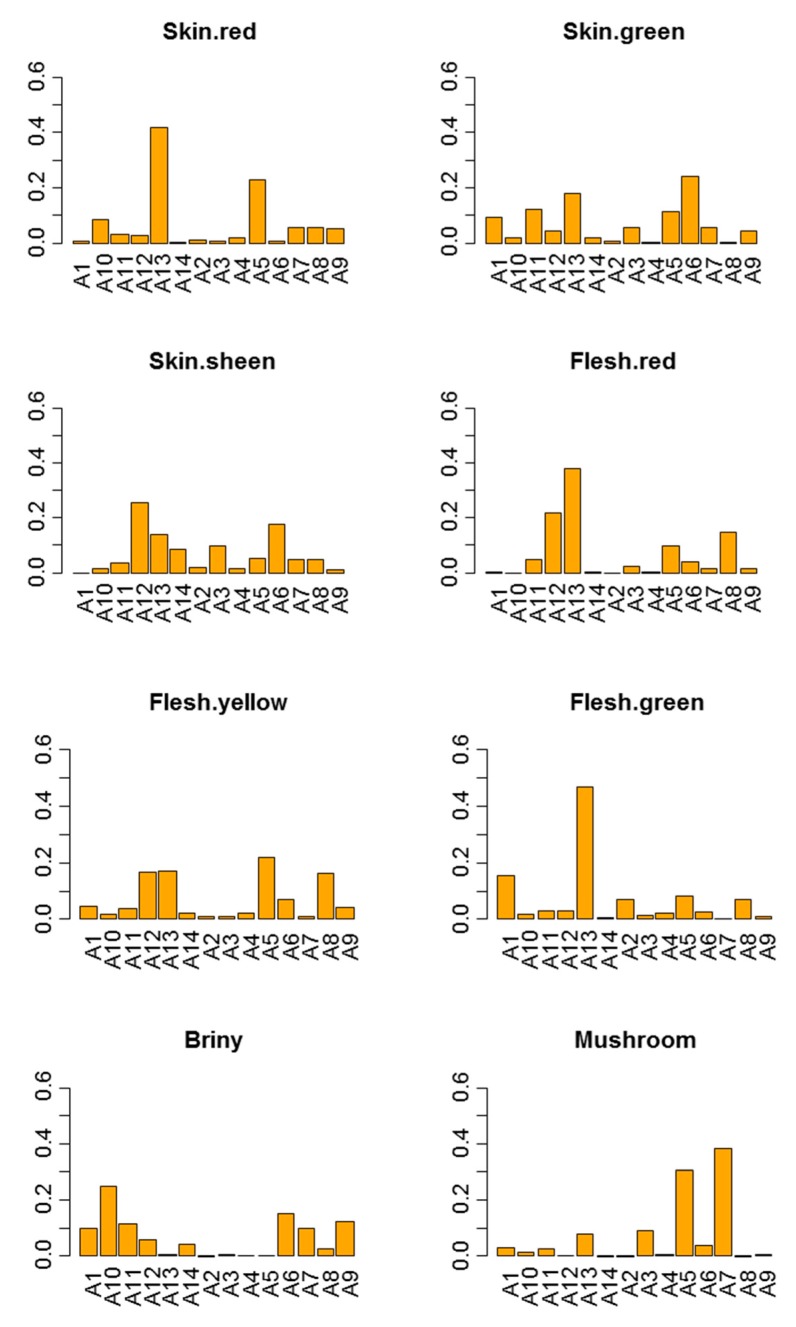
Panel performance. Panelist·session interaction. Contribution (coefficients) of panelists to the interaction for selected descriptors (skin red, skin green, skin sheen, flesh red, flesh yellow, flesh green, briny, and mushroom).

If significant, it means that one or more panelists do not similarly grade for all of the products from one session to another. There were several significant panelist·session interactions. Among the descriptors that contributed to discrimination, mushroom, oak barrel, cheesy smell, sourness, chewiness, bitterness, and saltiness had significant interactions (Table 1). The contribution of panelists to this interaction might also be evaluated by their respective coefficients, estimated as above-commented. Figure 4 shows examples.

Among the panelists that most contributed to the differences in scores between sessions according to descriptors, were: A13 for skin red, flesh red, and flesh green. Regarding other descriptors, A12 actively contributed to vinegar or A5 to natural fruity/floral, alcohol, and earthy soil (data not shown). However, most of the panelists had homogeneous contributions in most of the descriptors (skin green, skin sheen, flesh yellow, or briny, Figure 4). Moreover, no panelist showed a systematic trend for all descriptors, except a few of them, like A12 for skin sheen and flesh red or A7 for mushroom (Figure 4). Subsequently, the interaction was mainly due to the contribution of a reduced number of panelists (frequently only one) with limited influence on the panel repeatability.

**Figure 5:**
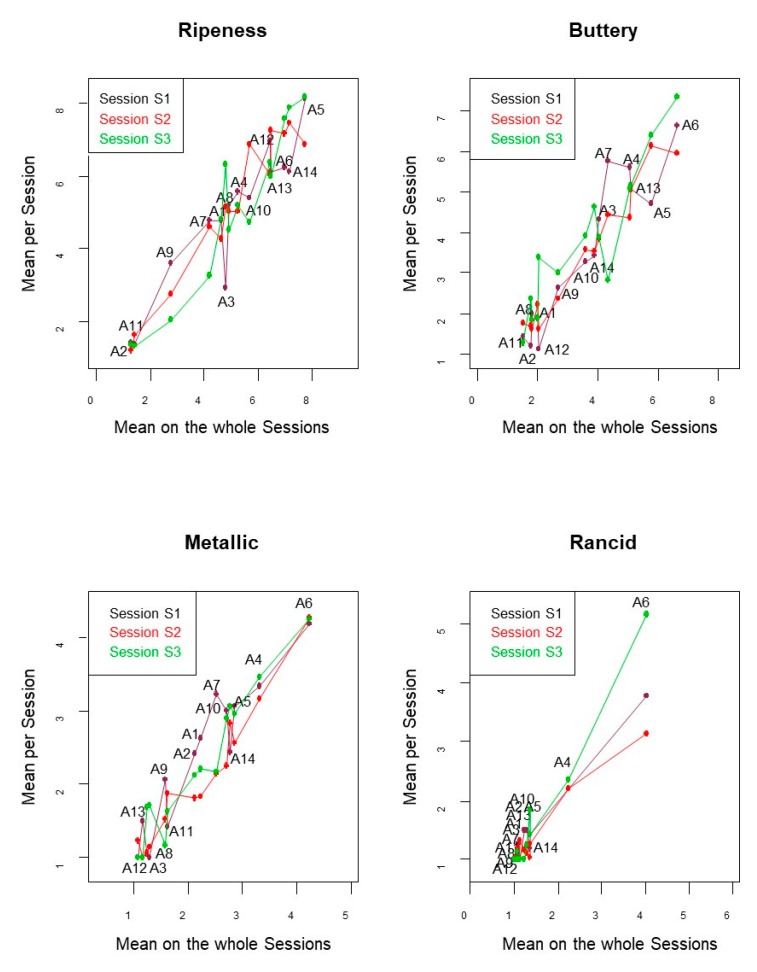
Panel performance. Panelist-session interaction. Means per session according to panelists over means of the whole sessions for selected descriptors (ripeness, buttery, metallic, and rancid).

The panelist·session interaction might also be presented as a plot of the mean per session over the mean on the whole sessions, according to panelists (Figure 5). Ideally, they should follow a line, regardless of sessions. In general, the panelists followed a similar trend over sessions (Figure 5 for some descriptors) with only punctual exceptions, like panelist A6 for rancid. Other cases were related to panelists A4, A12, and A8 for bitterness due to the abnormally low scores given by them (data not shown).

**Figure 6:**
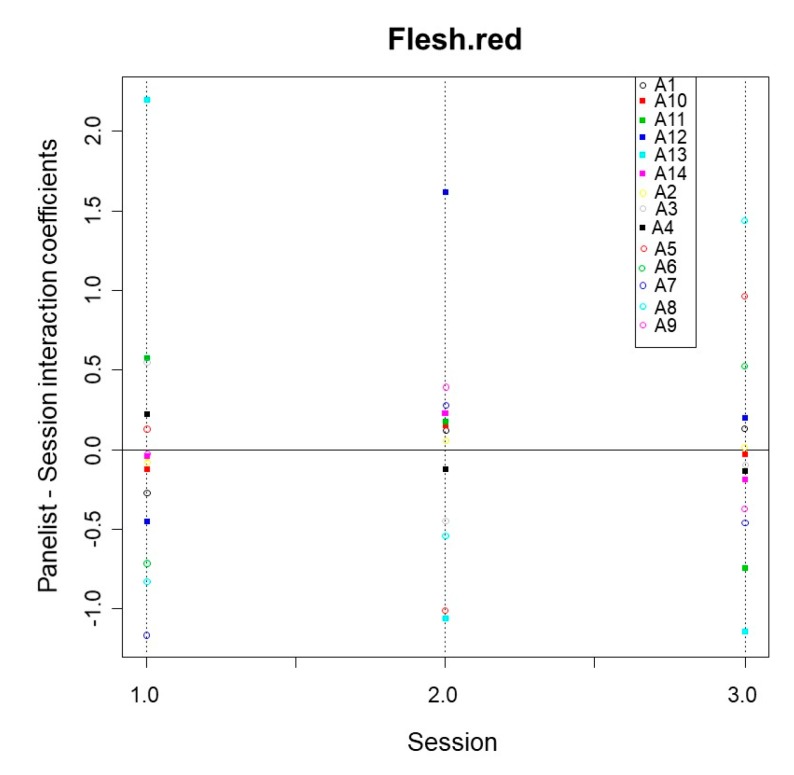
Panel performance. Panelist-session interaction. Detail of the coefficients through the three sessions for the flesh red descriptor.

Finally, the plot of the different coefficients over sessions is the most common evaluation of the panelist·session interaction (Figure 6, for flesh red as an example). In this case, the problems that could be observed are, again, of different ranking in successive sessions or different amplitude of scale over sessions. In Figure 6, panelist A13 assigned an excessive high score in the first session, while in the second session the score was low. Additionally, the amplitude of the scale for this descriptor was wider-spread in the first session than in the second. In saltiness, the situation was different, A12 had a very low contribution (coefficient) but the scale amplitude was similar among sessions; in firmness and fibrousness, panelist A13 was the only who had an excessive high score and, subsequently, a high contribution to the interaction, while, on the contrary, had low contribution on saltiness. Therefore, the analyses in detail of this interaction allowed for detecting some weakness of panel performance and lack of coherence in some panelist. Then, personalized training would be advisable.

### 3.3. Panelist Performance

When a panelist can discriminate among samples and is well repeatable and reproducible (that is, score the same product consistently and agrees with the rest of the panel), it is considered to be reliable according to Rossi [18]. There are several techniques for evaluating these panelist’s performance parameters. Tomic et al. [20] develop a series of graphs for easy visualisation of the sensory profiling data for performance. Kermit and Lengard Almli [19] mentioned consonance analysis with PCA, full ANOVA model and notation, assessor sensitivity, assessor reproducibility, or agreement test as appropriate to evaluate the assessor and panel performance. Lanza and Amoruso [17] mention the repeatability index (RI_t_) and deviation index (DI_t_) to evaluate how assessors perform against themselves over time and their performance with respect to the whole panel, respectively. In this work, the diverse tools that were proposed by Husson et al. [31] for studying the panelist work will be particularly followed.

#### 3.3.1. Discrimination Power of Each Panelist

The individual efficiency of panelists was evaluated with the model: score = sample + session. The *p*-values (Table 2) that are associated with the F-test of the sample effect on each panelist are, then, the appropriate parameter to measure this discrimination power. Their values, with rows and columns being sorted by the median estimated over them (Table 2), showed that most of the panelists were able to discriminate the black ripe table olive samples based on several of the descriptors that were developed by Lee et al. [9] and used later by López-López et al. [10]. Their efficiencies, in decreasing order, were: A14, A4, A2, A3, A6, A5, A8, A1, A12, A13, and A7, while only A11, A10, and A9 had not any discriminant power (Table 2). Skin green was the only descriptor that received an overall significant median; however, mouth coating, flesh red, briny, flesh green, or skin red were among the attributes most differently perceived in the samples (Table 2). On the contrary, soapy smell/medicinal, fishy smell, cheesy smell, alcohol, or metallic were among the most similarly perceived; however, this does not necessarily mean that the panelists were not able to differentiate samples, but that they were present in very low intensity or even completely absent (Table 2). There is controversy in the possible *p*-value that could be used as a cut off-level to consider one panelist acceptable. Stone et al. [32] proposed *p* ≥ 0.5, but the problem was that there were so many *p*-values below 0.5 when evaluating tea that almost any laboratory would retain them. Powers [33] pointed out that the real question was establishing the number of attributes with significant performance being necessary for a judge to be an acceptable assessor. However, no agreement on this aspect was achieved. In this work, in general, the panelists were not systematically excellent in all descriptors, but most of them were good at some descriptors (significant *p*-value), and their overall performance was reasonable; however, the behaviour of panelists A11, A10, and A9 should be, according to these results, candidates for possible further training or even removal from the panel if their performance will not sufficiently improve. Kermit and Lengard Almli [19] also identified an assessor with further need for training in attributes pea flavor, sweetness, fruity, and off flavor.

#### 3.3.2. Panelist Repeatability

The panelists’ repeatability is the ability to consistently score the same product for a given attribute [18] and was evaluated by the standard deviation (SD) of the measurements of a descriptor from each panelist on each sample. It was considered that, when the residual of the ANOVA model for each panelist and descriptor (Table 3) was ≤ 1.96 (*p* ≤ 0.95), the panelist scored the samples in a narrow range through the successive sessions and only panelists with residuals that were above this limit scored differently between sessions. In this work, there were no panelists who systematically graded the descriptors differently from one session to another (SD ≥ 1.96, in bold); however, several of them showed residuals above the limits for one to various descriptors, but not at a large distance. Therefore, in general, the panelists showed acceptable repeatability.

#### 3.3.3. Panelist Reproducibility

The panelist agreement with the panel, as associated to reproducibility [18], was assessed by the correlation between the panelists’ scores and the adjusted means of the panel (estimated by the ANOVA model) according to descriptors.

The procedure is similar to that used by Nyambaka et al. [30] to study the sensory changes in dehydrated cowpea leaves. The data are presented in a table, in which both panelists (in the column) and descriptors (in rows) are sorted from the highest to the lowest marginal median (Table 4). The panelists’ agreement with the panel (significant correlation, in black) were, in descending order of their medians, A6, A8, A14, A5, A1, A7, A13, A10, A9, A3, A2, A4, A12, and A11, while the negative correlation (in black and italic) was distributed more or less evenly, indicating opposed agreement with the panel (divergent behaviour). The inconsistence of some panelists when evaluating cowpea leaves was attributed to particular preferences of assessors [30] and could also be possible in table olives for some attributes, like firmness or fibrousness.

Overall, the descriptors that had the best agreement between panelists and panel, sorted by the median, were (in decreasing order of relationship) skin green, skin sheen, flesh red, firmness, flesh green, fibrousness, flesh yellow, and moisture release (Table 4). They were also among the descriptors with the most discriminant power. On the contrary, those with more discrepancies among the panelists were residual, artificial fruit/floral, metallic, rancid, sourness, or soapy smell/medical (Table 4), all of them with no discriminant influence.

These results show that the overall behaviour of the panelists was reasonable, although there was still margin for some improvement in their performance, particularly regarding those panelists with strongly opposed correlation to the mean of the panel. Alternatively, they could be candidates for further rejection.

Lanza and Amoruso [17] used line plot according to the attribute and deviation index (DI_t_) to evaluate the agreement between panelists and whole panel. Their results are in line with those described above, since they also found some panelists who clearly deviated from the consensus. According to these authors, this type of results helps the panel leader to identify repeatability problems of specific assessors as compared to the whole panel and correct the deviation by the corresponding training.

### 3.4. Multivariate Study of Panelists and Panel

#### 3.4.1. Clustering

**Figure 7:**
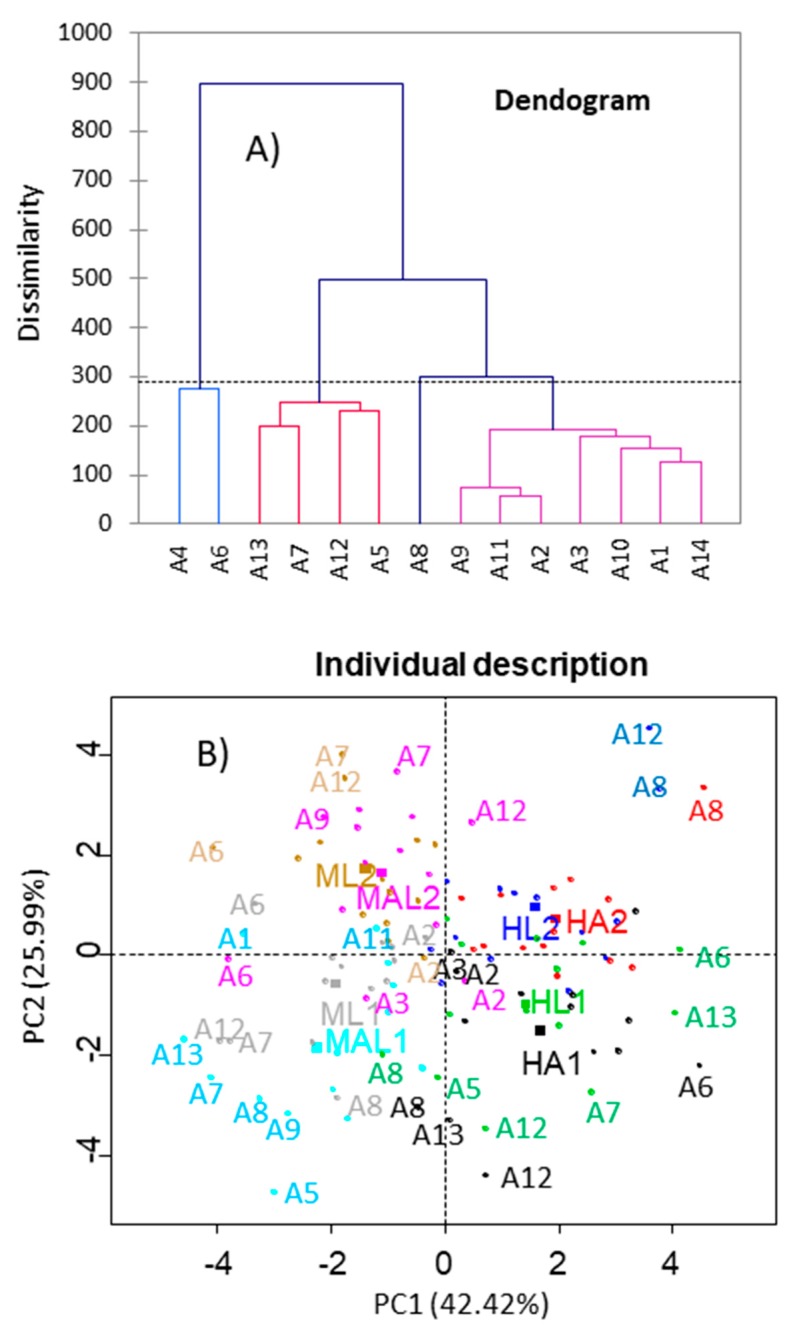
Panelist performance as assessed by multivariate analysis. (**A**) Clustering of panelists according to their performance. (**B**) Projection of panelists’ loads (individual description) and samples’ scores onto the first two Principal Components.

A first multivariate approach of the similarity among panelists was achieved by hierarchical clustering analysis based on the scores given to the sample descriptors by each of them. The study was performed in XLSTAT, while using Wards’ aggregation criterion [28]. Three groups of panelists were formed when comparing the panelists’ behaviour (Figure 7A). The greatest dissimilarity was found between the group that was formed by A4 and A6 with respect to the other panelists. The dissimilarity within the groups of other panelists was sensibly lower, leading to three groups. Two of them were composed of four and seven panelists, while the third only included panelist A8, who had a peculiar behaviour. Therefore, in this case, the cluster analysis, which considers the overall panelist performance, showed that the panelists followed a somewhat similar trend when evaluating the black ripe olive samples, but not reveal their peculiarities. In line with this result, the hierarchical classification is more usually applied for the classification of products or studying the association among descriptors. Francois et al. [28] used this technique for assessing the astringency of different beers while Pense-Lheritier et al. [29] applied it to link the sensory changes induced by the addition of drugs to different beverages. Alasalvar et al. [6] found similarity among the flavor of natural and roasted Turkish hazelnut cultivars. Clustering was also used to segregate different consumers segments according to their overall liking scores [34].

#### 3.4.2. Panelist Reproducibility

The multivariate study of the agreement among panelists and the whole panel [18], while using bootstrapping, was made in SensoMiner, by considering the results of a virtual panel that was obtained by taking successive samples (500 simulations) from the real data and applying Principal Component Analysis. Only two eigenvalues ≥1 were found and they accounted for ~42 and 26% of the variance, respectively. The analysis was made while using the function panelipse·session. The resampling technique has been described in detail elsewhere [31].

The closeness of the whole panel and panelists’ answers was evaluated by projecting them onto the first two PCs. A PCA on the consensus allows for visualizing the strength of the consensus and the global discrimination of the products; besides, treatments identification shows the observed differences between the products [35]. In this work, the distance from each panelist to the situation of the corresponding sample assessed the agreement between the whole panel (squares symbols and different colours for the samples) and the panelists’ acronyms (associated to samples by circle symbols using the same colours) (Figure 7B).

PC1 was highly efficient for segregating samples from Manzanilla (on the left) and Hojiblanca (on the right) and it could be associated to cultivar, while PC2 was able to distinguishing samples as a function of growing area and storage. In general, the projections of panelists for each sample were situated around that of the whole panel (sample associated to the same colour); although, there were some of them far for their respective samples. The discrepant panelists were (as identified by the corresponding acronyms) the same already mentioned in previous sections, mainly: A12, A8 for HL2; A8 for HA2; A13, A12, A8 and A6 for HA1; A12, A7, A9, A6, A3, and A2 for MAL2; A12, A7, A6, and A2 for ML2; A13, A11, A9, A8, A7, A5, and A1 for MAL1; and, A12, A8, A7, A6, and A2 for ML1. The panelist who scored the samples differently more times was A12, followed by A8, A7, and A6. Lower discrepancies were observed for A2, A9, A13, A3, and A5. However, they represent just a few cases of divergences, while most of the panelists’ scores are jointly distributed around their corresponding samples. Additionally, panelists had greater ability (closeness to the sample average) to evaluate long stored Hojiblanca samples (HL2 and HA2) than any other sample. In conclusion, this plot has identified the panelists who will require particular training, but the performance of the others will also benefit from training. Our results are in agreement to those that were presented by Tomic et al. [21], who also found underperformance panelists and emphasized the need for a detailed study of their behavior while using the established statistical methods for the evaluation. Lanza and Amoruso [17] studied the performance of panelist against the whole panel using Eggsshell plots, concluding that there were also a few panelists that ranked some of the descriptors quite differently from the consensus, while there was a good agreement in others, like hardness.

#### 3.4.3. Panel Repeatability

##### Study by Variables Projection on the Correlation Circle According to Sessions

**Figure 8:**
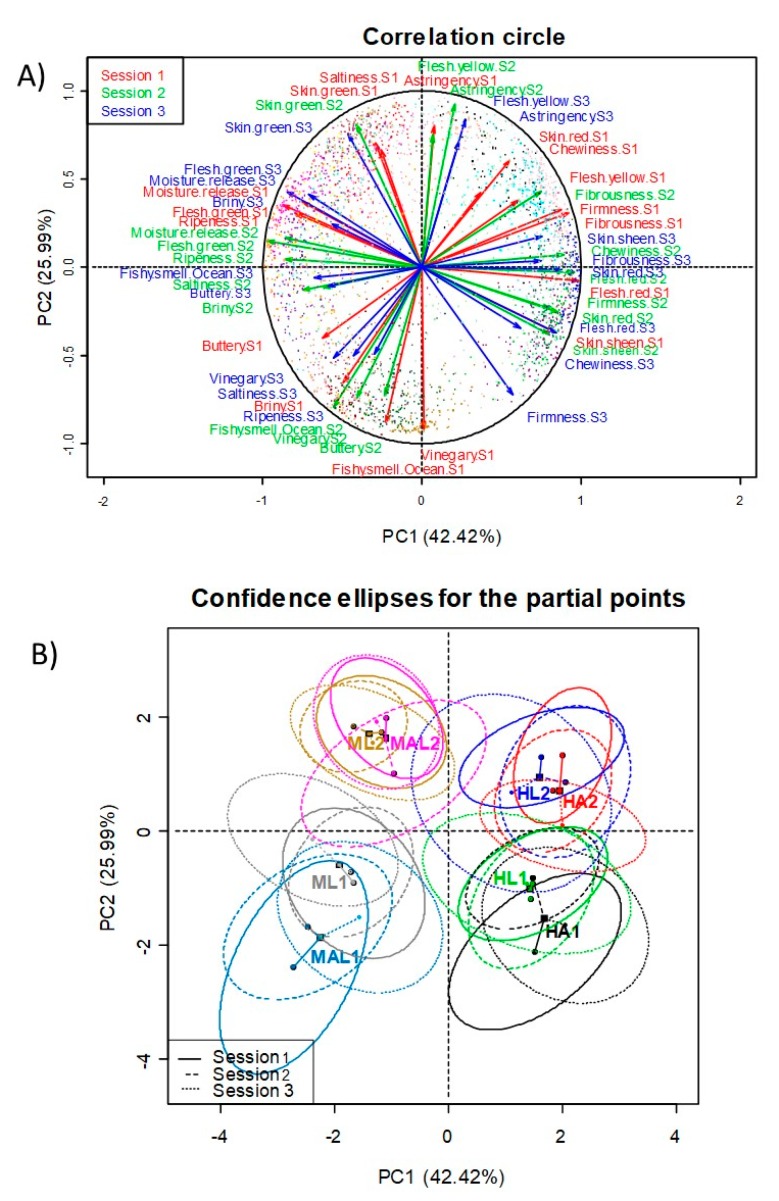
Panel repeatability as assessed by multivariate analysis, using bootstrapping. (**A**) Projection of the descriptors ‘loads on the correlation circle onto the first two Principal Components, and (**B**) Projection of the samples’ scores and confidence ellipses according to sessions onto the first two Principal Components.

The analysis was carried out using the virtual panel described above [31]. A first approach of the panel repeatability was observed by projecting the descriptors (only those more relevant, contribution >0.20) onto the first two PC according to sessions. Close situations of descriptors in the correlation circle for the different sessions indicate good repeatability. The panel was particularly repeatable among sessions for some descriptors, like skin green, astringency, flesh green, moisture release, fibrousness, flesh red, skin sheen, or flesh yellow. However, others had sensible distances from one session to another, like fishy smell/ocean, saltiness, or chewiness (Figure 8A). The interpretation of the relationships among variables is not straightforward due to these oscillations on the variables’ projections. Nevertheless, it is possible to establish overall associations, mainly in those variables with high repeatability among sessions. For example, firmness, fibrousness, or chewiness are opposed to moisture release, ripeness, or flesh green. Additionally, those black ripe olives with high astringency could also present flesh yellow or skin green notes, but low vinegar or ripeness scores.

Galán Soldevilla et al. [14] associated bitter, sour, and wood with *Green*, *Cured*, and *Traditional Aloreña de Málaga* table olives, respectively. In black ripe olives, discrimination among the samples from different origins was mainly based on the 2nd and 3rd PCs, which were the components linked to aroma and flavour characteristics; however, the more linear behaviour of panelists was related to a textural dimension that was strongly connected to PC1 [9]. Kinesthetic sensations were also critical for the segregation between defected and un-defected samples by PCA [12].

##### Study by Sample Projections According to Sessions

The analysis was also carried out using the virtual panel described above. In this case, the median scores of the virtual panel perception of the samples (the same of the real panel) were projected onto the plane of the two first PCs according to sessions. Subsequently, 95% of the closest points of the generated cloud of points were used to draw their confidence ellipses (*p*-value = 0.05), which were built according to the procedure that was described by Husson et al. [31] (Figure 8B). The repeatability of the panel to the session can be assessed by the displacement of the sample centres. In general, the separation between the sample centres due to session was limited, indicating a good panel agreement between sessions, which is also corroborated by the overlapping of their confidence ellipses. Incidentally, the plot also indicates that the long stored fruits showed lower dispersion by sessions than the just processed fruits (one-month storage).

## 4. Conclusions

Usually, the study of the panel performance is a previous, but superficial, task during the sensory evaluation of products. However, a detailed investigation of the panel and panelist performance is a convenient tool to uncover the details of their evaluation. In this work, such study allowed for the assessment of the panel performance as a whole, as well as detecting the panelist with the lowest discriminant power, those that have interpreted the scale in a different way than the panel and, therefore, require further training or even discovery that the stored black ripe olive products are more similarly perceived by the panelists over sessions. Besides, the study identified the descriptors of hard evaluation (skin green, vinegar, bitterness, or natural fruity/floral). Therefore, panelists would require particular training on them or, in case of not reaching the appropriate level of discrimination, be replaced by some other/s with higher sensitivity. In summary, the work has confirmed that such studies are an essential tool for the appropriate panel control and training, which should be a permanent concern of the panel leader.

## Figures and Tables

**Table 1:** Overall panel performance as assessed by analysis of variance (ANOVA) sorted by sample *p*-values, including main effects, and interactions. Panelist, session, and their interactions were considered as random, while the sample was studied as fixed factor/variable.

Sensory Attribute	Sample	Panelist	Session	Sample·Panelist	Sample·Session	Panelist·Session	Median
Skin green	**2.792 × 10**^**−10**^	**9.069 × 10**^**−26**^	2.177 × 10^−1^	**2.404 × 10**^**−8**^	6.211 × 10^−1^	9.423 × 10^−2^	**4.712 × 10**^**−2**^
Flesh green	**1.720 × 10**^**−9**^	**2.186 × 10**^**−15**^	2.377 × 10^−1^	**2.602 × 10**^**−4**^	7.443 × 10^−1^	6.387 × 10^−1^	1.190 × 10^−1^
Skin sheen	**1.900 × 10**^**−6**^	**1.742 × 10**^**−28**^	2.497 × 10^−1^	**1.011 × 10**^**−4**^	6.362 × 10^−1^	2.689 × 10^−1^	1.249x 10^−1^
Flesh red	**8.603 × 10**^**−6**^	**2.654 × 10**^**−34**^	6.326 × 10^−1^	**1.796 × 10**^**−9**^	5.180 × 10^−1^	**3.250 × 10**^**−3**^	**1.629 × 10**^**−3**^
Firmness	**1.033 × 10**^**−4**^	**4.141 × 10**^**−35**^	3.320 × 10^−1^	**1.881 × 10**^**−3**^	9.960 × 10^−2^	**3.305 × 10**^**−2**^	**1.747 × 10**^**−2**^
Fibrousness	**9.292 × 10**^**−4**^	**2.088 × 10**^**−39**^	4.165 × 10^−1^	**1.397 × 10**^**−3**^	4.263 × 10^−1^	**9.392 × 10**^**−3**^	**5.394 × 10**^**−3**^
Flesh yellow	**1.328 × 10**^**−2**^	**7.265 × 10**^**−20**^	2.752 × 10^−1^	**1.752 × 10**^**−4**^	2.046 × 10^−1^	2.332 × 10^−1^	1.090 × 10^−1^
Skin red	**1.760 × 10**^**−2**^	**3.535 × 10**^**−51**^	6.585 × 10^−1^	**1.511 × 10**^**−10**^	2.695 × 10^−1^	1.342 × 10^−1^	7.590 × 10^−2^
Vinegary	**1.833 × 10**^**−2**^	**3.683 × 10**^**−30**^	1.613 × 10^−1^	**1.794 × 10**^**−3**^	6.612 × 10^−2^	**2.908 × 10**^**−2**^	**2.370 × 10**^**−2**^
Moisture release	**2.978 × 10**^**−2**^	**4.515 × 10**^**−33**^	9.058 × 10^−1^	**9.558 × 10**^**−6**^	5.027 × 10^−1^	6.828 × 10^−2^	**4.903 × 10**^**−2**^
Fishy smell/Ocean	**3.060 × 10**^**−2**^	**1.312 × 10**^**−12**^	3.342 × 10^−1^	3.912 × 10^−1^	7.507 × 10^−1^	7.249 × 10^−1^	3.627 × 10^−1^
Saltiness	**3.117 × 10**^**−2**^	**1.680 × 10**^**−48**^	3.191 × 10^−1^	**2.575 × 10**^**−3**^	**3.940 × 10**^**−3**^	**1.068 × 10**^**−3**^	**3.258 × 10**^**−3**^
Astringency	2.232 × 10^−1^	**1.599 × 10**^**−45**^	9.491 × 10^−1^	**6.062 × 10**^**−14**^	6.593 × 10^−1^	1.447 × 10^−1^	1.839 × 10^−1^
Ripeness	2.614 × 10^−1^	**1.586 × 10**^**−44**^	8.095 × 10^−1^	**4.834 × 10**^**−5**^	6.614 × 10^−1^	**6.012 × 10**^**−3**^	1.337 × 10^−1^
Soapy smell/Medicinal	2.636 × 10^−1^	**2.478 × 10**^**−51**^	4.467 × 10^−1^	4.560 × 10^−1^	6.792 × 10^−1^	**2.451 × 10**^**−2**^	3.552 × 10^−1^
Bitterness	2.710 × 10^−1^	**4.434 × 10**^**−38**^	2.556 × 10^−1^	5.075 × 10^−1^	1.940 × 10^−1^	**2.364 × 10**^**−3**^	2.248 × 10^−1^
Chewiness	3.334 × 10^−1^	**1.538 × 10**^**−39**^	7.989 × 10^−1^	**4.862 × 10**^**−11**^	2.964 × 10^−1^	**2.947 × 10**^**−3**^	1.497 × 10^−1^
Briny	3.567 × 10^−1^	**1.708 × 10**^**−37**^	7.944 × 10^−1^	**4.671 × 10**^**−6**^	8.067 × 10^−1^	**2.152 × 10**^**−2**^	1.891 × 10^−1^
Natural fruity/Floral	4.102 × 10^−1^	**2.781 × 10**^**−30**^	1.840 × 10^−1^	**4.931 × 10**^**−3**^	6.946 × 10^−1^	3.302 × 10^−1^	2.571 × 10^−1^
Rancid	4.867 × 10^−1^	**1.815 × 10**^**−41**^	3.093 × 10^−1^	2.110 × 10^−1^	6.873 × 10^−1^	**2.669 × 10**^**−2**^	2.601 × 10^−1^
Nutty	4.892 × 10^−1^	**4.653 × 10**^**−26**^	3.041 × 10^−1^	**3.637 × 10**^**−3**^	2.203 × 10^−1^	**1.108 × 10**^**−2**^	1.157 × 10^−1^
Buttery	5.223 × 10^−1^	**7.225 × 10**^**−42**^	3.749 × 10^−1^	**1.292 × 10**^**−9**^	4.572 × 10^−1^	**6.488 × 10**^**−3**^	1.907 × 10^−1^
Oak barrel	5.496 × 10^−1^	**4.336 × 10**^**−44**^	3.501 × 10^−1^	**7.740 × 10**^**−3**^	9.681 × 10^−2^	**2.796 × 10**^**−6**^	5.227 × 10^−2^
Metallic	5.778 × 10^−1^	**1.374 × 10**^**−24**^	1.115 × 10^−1^	1.010 × 10^−1^	**5.508 × 10**^**−6**^	8.859 × 10^−1^	1.062 × 10^−1^
Alcohol	6.690 × 10^−1^	**9.806 × 10**^**−60**^	8.765 × 10^−2^	2.337 × 10^−1^	7.464 × 10^−1^	1.730 × 10^−1^	2.033 × 10^−1^
Mushroom	6.795 × 10^−1^	**5.867 × 10**^**−26**^	4.712 × 10^−1^	**3.910 × 10**^**−6**^	1.256 × 10^−1^	**6.242 × 10**^**−7**^	6.280 × 10^−2^
Mouth coating	6.925 × 10^−1^	**2.358 × 10**^**−56**^	7.926 × 10^−1^	**5.014 × 10**^**−17**^	2.719 × 10^−1^	**1.033 × 10**^**−3**^	1.365 × 10^−1^
Sourness	7.219 × 10^−1^	**1.227 × 10**^**−24**^	6.145 × 10^−1^	**1.206 × 10**^**−2**^	8.290 × 10^−1^	**8.804 × 10**^**−4**^	3.133 × 10^−1^
Earthy/Soil	7.335 × 10^−1^	**1.713 × 10**^**−28**^	2.620 × 10^−1^	2.050 × 10^−1^	8.907 × 10^−2^	3.771 × 10^−1^	2.335 × 10^−1^
Artificial fruity/Floral	8.387 × 10^−1^	**8.279 × 10**^**−37**^	2.036 × 10^−1^	**1.018 × 10**^**−4**^	9.372 × 10^−1^	8.575 × 10^−1^	5.211 × 10^−1^
Residual	8.937 × 10^−1^	**4.326 × 10**^**−43**^	1.212 × 10^−1^	**1.784 × 10**^**−9**^	6.901 × 10^−1^	9.118 × 10^−2^	1.062 × 10^−1^
Cheesy smell	9.075 × 10^−1^	**2.015 × 10**^**−27**^	6.441 × 10^−1^	**8.088 × 10**^**−3**^	3.692 × 10^−1^	**2.652 × 10**^**−4**^	1.886 × 10^−1^
Gassy smell	9.389 × 10^−1^	**3.652 × 10**^**−33**^	5.786 × 10^−1^	1.249 × 10^−1^	9.484 × 10^−1^	7.670 × 10^−1^	6.728 × 10^−1^

Note: Significant values at *p* ≤ 0.05 are indicated in bold.

**Table 2:** Discriminant power of panelists as assessed by the *p*-value of the F test.

	A14	A4	A2	A3	A6	A5	A8	A1	A12	A13	A7	A11	A10	A9	Median
**Skin green**	**0.0007**	**0.0119**	**0.0011**	**0.0010**	**0.0016**	0.0687	**0.0002**	0.1243	**5.2 × 10**^**−7**^	**0.0402**	0.0655	0.5177	0.6086	0.1772	**0.0261**
**Mouth coating**	**0.0491**	**0.0188**	0.2445	**0.0023**	**0.0162**	**0.0063**	**4.8 × 10**^**−7**^	0.0927	0.7896	**0.0042**	0.2880	0.4278	0.2185	0.8250	0.0709
**Flesh red**	**0.0103**	**0.0294**	0.448	**0.0019**	**3.9 × 10**^**−6**^	**0.0073**	0.6432	0.3536	**0.0409**	0.1384	**2.4 × 10**^**−5**^	0.1661	0.4706	0.1965	0.0897
**Briny**	0.0912	**0.0490**	**0.0074**	0.3023	0.0138	0.1940	0.0679	0.5585	**0.0370**	0.1132	0.4739	0.2358	0.1762	**0.0419**	0.1022
**Flesh green**	0.0564	**0.0049**	0.0885	0.0792	0.0001	0.2135	0.1192	0.1599	**0.0240**	0.1850	5.3 × 10^−7^	0.3497	0.1588	0.1216	0.1039
**Skin red**	**6.7 × 10**^**−6**^	0.1042	**0.0225**	**1.0 × 10**^**−6**^	**0.0063**	0.5103	0.1245	0.3750	**0.0251**	**0.0046**	0.2455	0.0883	0.4822	0.2204	0.0963
**Residual**	0.1602	**0.0005**	0.4853	0.1151	**0.0480**	0.0976	**4.9 × 10**^**−10**^	0.0514	0.5184	0.6976	0.3556	0.2728	0.7313	0.0727	0.1376
**Oak barrel**	0.1427	**0.0006**	0.9411	0.1950	0.2170	0.8693	0.4858	**0.0194**	0.1548	0.2937	0.4778	0.1655	0.1373	0.1540	0.1802
**Fibrousness**	0.1065	**0.0220**	**6.4 × 10**^**−5**^	0.3311	0.1991	0.3859	**0.0055**	0.1004	**0.0410**	0.5118	0.0525	0.6702	0.4188	0.6076	0.1528
**Ripeness**	0.1602	**0.0005**	0.4853	0.4853	**0.0480**	0.0976	**4.9 × 10**^**−10**^	0.0514	0.5184	0.6976	0.3556	0.2728	0.7313	0.0727	0.1376
**Firmness**	0.1345	**0.0402**	**0.0444**	**0.0022**	0.1130	0.7759	0.1552	0.4107	0.2639	0.1660	0.0009	0.3727	0.2481	0.8867	0.1606
**Skin sheen**	0.2700	0.2860	**0.0323**	**0.0003**	**0.3672**	0.0109	0.0269	**7.7 × 10**^**−5**^	0.1841	**0.0009**	0.6877	0.1887	0.1489	0.8764	0.1665
**Flesh yellow**	0.1231	**0.0116**	0.081	0.1643	0.7031	0.2925	0.0878	0.5186	**0.0442**	0.5177	**6.6 × 10**^**−5**^	0.3582	0.0754	0.2687	0.1437
**Bitterness**	**0.0100**	0.6474	**0.0056**	0.2079	0.5114	0.7813	0.2312	0.0501	0.2547	0.2341	0.9994	0.2390	0.1831	0.5097	0.2365
**Astringency**	0.5481	**0.0240**	**0.0374**	0.2538	0.6887	0.2064	**1.5 × 10**^**−8**^	**0.0159**	0.1436	0.7140	0.0508	0.9816	0.6175	0.4225	0.2301
**Moisture release**	0.1299	0.2333	0.2658	**0.0004**	**0.0052**	0.6328	**0.0480**	0.3207	0.0604	0.0330	0.102	0.5576	0.8638	0.2983	0.1816
**Nutty**	**0.0392**	0.0793	0.3598	0.2546	0.1677	0.0824	0.2438	0.2683	0.4706	0.8304	0.4658	0.1908	0.3928	0.1083	0.2492
**Buttery**	**7.6 × 10**^**−7**^	0.2047	0.2191	**6.1 × 10**^**−6**^	0.6094	0.1268	0.8115	**0.0356**	0.2662	0.1992	0.0634	0.5658	0.3017	0.9222	0.2119
**Mushroom**	0.8962	**0.0003**	0.6540	**0.0006**	0.6046	0.3799	0.7030	**0.0476**	0.4706	0.5158	**0.0077**	0.1854	0.5196	0.136	0.4253
**Chewiness**	**0.0003**	0.4448	0.7152	**0.0156**	0.2636	0.6584	**6.5 × 10**^**−9**^	**0.0003**	**0.0422**	0.2922	0.1441	0.6937	0.9195	0.6294	0.2779
**Sourness**	0.2963	0.6765	0.6530	0.4706	0.0155	0.1844	0.4783	0.2397	0.3977	0.2461	0.3597	0.8106	0.0778	0.7095	0.3787
**Saltiness**	0.5401	0.6428	0.4611	0.1292	0.0715	0.2307	**0.0075**	0.0147	0.1889	0.9875	0.7995	0.7577	0.4706	0.1714	0.3459
**Vinegary**	0.1315	0.6339	0.3024	0.3830	0.2282	0.1108	0.4706	0.3442	0.0716	0.5815	0.3609	0.4651	0.4706	0.5146	0.3719
**Nat. fruity/floral**	0.0598	0.7439	0.7778	**0.0112**	0.4959	0.4959	0.4706	0.3475	0.6836	0.2703	0.6252	0.3119	0.0554	0.7819	0.4091
**Earthy soil**	0.3278	**0.0136**	0.1162	0.6321	0.9603	0.2985	0.4706	0.561	0.4706	0.3367	0.5492	0.4013	0.5284	0.1496	0.4360
**Rancid**	0.09477	0.2697	0.2386	0.5123	0.3915	0.6038	0.4706	0.3878	0.4706	0.4822	0.5793	0.1436	0.5866	0.6142	0.4706
**Art. fruity/floral**	**4.4 × 10**^**−5**^	0.4568	0.3301	0.5810	0.0916	**0.0457**	**0.0279**	0.2441	0.4706	0.0880	0.4715	0.4706	0.4876	0.7257	0.3934
**Metallic**	0.2645	0.5882	**0.0167**	0.4980	0.5781	0.8309	0.5770	0.3776	0.4706	0.4706	0.3236	0.3508	0.4074	0.5429	0.4706
**Alcohol**	0.3289	0.4027	0.1303	0.5559	0.7270	0.2080	0.5836	0.4118	0.4706	0.3147	0.3841	0.4706	0.4706	0.7604	0.4412
**Cheesy smell**	0.5312	0.5468	0.1270	0.6647	0.3881	0.7116	0.4706	0.3594	0.4706	0.7706	0.1850	0.5121	0.0275	0.7174	0.4914
**Fishy smell**	0.7693	0.1763	0.1578	0.5570	0.2083	0.7899	0.4706	0.3009	0.4706	**0.0289**	0.4288	0.1892	0.7001	0.5644	0.4497
**Soapy smell/med**	0.6524	0.5964	0.1828	0.5373	0.7019	0.6411	0.6237	0.8154	0.4706	**0.0145**	0.4853	0.5051	0.7512	0.4998	0.5669
**Median**	0.1299	0.1763	0.1828	0.1950	0.2170	0.2307	0.2312	0.2583	0.2662	0.2703	0.3556	0.3582	0.4706	0.5097	0.2448

Note: Significant values at *p* ≤ 0.05 are indicated in bold.

**Table 3:** Panelist repeatability as assessed by the ANOVA residuals according to descriptors.

	A1	A10	A11	A12	A13	A14	A2	A3	A4	A5	A6	A7	A8	A9
**Skin red**	1.63	**2.27**	0.88	1.85	1.91	1.03	0.60	1.06	0.94	**2.74**	1.69	**2.06**	1.41	1.03
**Skin green**	1.50	**2.06**	1.26	1.47	1.96	1.37	0.31	1.30	0.73	1.75	1.23	**2.02**	1.03	**2.42**
**Skin sheen**	0.98	1.57	1.27	**3.20**	1.61	1.15	0.51	1.43	1.03	1.39	1.82	1.81	0.77	1.63
**Flesh red**	1.74	0.06	1.41	**2.89**	**3.10**	1.23	0.84	1.64	0.94	1.58	1.11	1.61	1.86	1.27
**Flesh yellow**	1.22	1.90	1.38	**2.77**	1.16	1.16	0.67	0.21	0.67	1.55	**2.41**	0.78	1.75	0.91
**Flesh green**	1.76	**2.05**	1.46	**2.76**	**2.64**	1.65	0.54	1.06	0.88	**2.88**	1.40	1.11	1.85	**2.08**
**Briny**	1.37	1.58	1.09	**2.32**	1.34	0.91	0.46	1.00	0.39	1.41	1.45	1.74	1.27	1.75
**Mushroom**	0.58	0.91	1.06	0.09	1.35	1.09	0.75	1.13	0.53	1.08	**2.19**	1.65	1.41	0.88
**Earthy soil**	0.78	1.66	**2.00**	0.09	0.98	0.29	0.54	0.68	0.86	1.21	1.86	0.59	<0.01	0.77
**Oak barrel**	0.41	1.93	0.65	1.33	1.03	0.36	0.89	0.53	0.59	1.63	1.88	1.06	1.93	0.79
**Nutty**	0.21	1.19	0.80	0.10	0.64	0.40	0.54	0.77	0.54	0.51	1.07	0.96	**2.06**	1.33
**Artificial fruity/floral**	0.18	0.93	***0.02***	0.12	0.72	0.36	0.59	0.15	0.78	1.14	1.19	1.19	0.79	0.96
**Natural fruity/floral**	1.13	1.74	0.07	1.03	1.06	0.87	0.87	0.79	0.80	1.61	1.95	1.50	**<0.01**	1.05
**Vinegary**	0.24	***<0.01***	0.28	1.55	1.20	0.38	0.53	0.39	0.53	**2.31**	**1.98**	0.59	0.41	0.61
**Alcohol**	0.20	0.13	0.07	0.14	0.61	0.67	0.55	0.26	0.60	**2.03**	1.73	1.09	1.04	1.00
**Fishy smell/ocean**	0.48	0.85	0.15	0.14	0.96	1.08	0.31	0.38	0.84	1.37	1.46	1.71	0.35	1.58
**Cheese smell**	0.44	1.00	0.09	0.20	0.86	0.09	0.29	0.09	0.47	0.76	1.39	0.55	***0.02***	0.15
**Sourness**	0.14	0.56	0.53	1.28	0.35	1.89	0.14	0.20	0.70	**1.98**	0.84	1.65	0.65	0.28
**Bitterness**	0.80	1.06	0.30	1.27	1.54	0.87	0.42	0.55	0.87	**1.99**	1.07	**2.73**	1.59	0.37
**Saltiness**	0.78	0.13	0.65	1.75	0.76	0.27	0.30	0.40	0.86	1.19	1.19	**2.35**	1.33	0.93
**Ripeness**	1.27	**2.81**	0.52	**2.12**	1.95	1.73	0.45	0.98	0.73	1.43	1.49	**2.07**	1.69	1.04
**Buttery**	0.71	**2.26**	0.68	1.41	1.71	0.91	0.53	1.18	0.77	1.09	1.87	1.57	1.26	**2.12**
**Metallic**	1.62	1.37	0.54	0.25	0.82	1.45	0.52	1.09	0.95	1.84	**2.00**	1.69	0.82	0.80
**Rancid**	0.36	0.73	***0.04***	***<0.01***	0.45	0.56	0.37	0.33	0.74	0.70	**2.01**	0.26	***<0.01***	***0.05***
**Soapy smell/medicinal**	0.30	1.37	0.46	0.42	1.21	0.05	0.30	0.85	0.81	0.91	**2.50**	1.14	0.66	0.71
**Gassy smell**	0.12	0.59	0.05	0.71	***<0.01***	**<0.01**	0.12	0.47	0.70	0.12	1.41	0.13	***0.022***	0.07
**Firmness**	1.29	1.36	0.64	1.12	**2.36**	1.68	0.38	1.14	0.82	1.85	1.83	1.10	1.03	1.01
**Fibrousness**	1.02	0.77	0.62	1.25	**2.02**	1.43	0.25	1.51	0.71	1.14	1.56	1.35	1.53	1.17
**Moisture release**	1.36	2.02	0.61	1.20	1.85	1.34	0.29	1.01	0.67	0.99	1.41	1.32	1.03	1.42
**Mouth coating**	0.85	1.73	0.60	0.65	1.30	1.32	0.52	1.20	0.64	0.72	1.15	1.00	0.84	0.92
**Chewiness**	0.75	1.46	0.64	1.10	1.94	1.11	0.36	1.10	1.26	1.36	1.73	1.04	0.84	0.92
**Astringency**	0.05	1.60	0.53	1.19	**1.99**	0.07	0.12	0.91	0.62	0.54	1.37	1.45	0.91	0.10
**Residual**	0.62	**2.14**	0.13	0.13	1.41	1.89	1.36	0.34	0.95	0.82	1.35	1.28	1.04	0.55

Notes: Significant higher values at *p* ≤ 0.05 are indicated in bold while an agreement is showed as bold and italic.

**Table 4:** Panelist agreement with panel as assessed by the correlation coefficient.

	A6	A8	A14	A5	A1	A7	A13	A10	A9	A3	A2	A4	A12	A11	Median
**Skin green**	0.674	0.828	0.792	0.810	0.660	0.603	**0.932**	0.547	0.814	0.808	0.853	0.874	0.791	**0.860**	0.800
**Skin sheen**	0.762	0.787	0.840	**0.962**	**0.809**	**0.869**	0.677	0.820	0.050	0.220	0.092	0.883	0.762	0.323	0.744
**Flesh red**	**0.907**	**0.904**	0.499	0.841	0.667	**0.956**	0.190	***−0.322***	0.816	0.268	0.759	0.524	0.722	0.420	0.695
**Firmness**	0.335	0.678	0.600	0.296	0.754	0.758	0.789	0.696	0.500	0.216	0.720	**0.875**	0.335	0.803	0.687
**Flesh green**	**0.960**	0.601	0.152	0.198	0.752	0.550	0.727	0.615	0.505	0.751	0.344	0.764	0.097	0.572	0.637
**Fibrousness**	0.644	0.639	0.152	0.198	0.752	0.550	0.727	0.615	0.505	0.751	0.344	0.833	0.965	***−0.658***	0.577
**Flesh yellow**	0.309	0.673	0.293	0.529	**−*****0.635***	0.598	0.801	0.556	0.653	0.306	0.649	0.538	**−*****0.226***	0.504	0.558
**Moist. release**	0.781	0.425	0.579	0.588	0.667	0.605	0.355	***−0.078***	0.653	0.306	0.649	0.538	**−*****0.226***	0.504	0.558
**Fishy smell**	***−0.018***	0.777	**0.885**	0.434	0.336	0.366	0.723	0.216	0.650	**0.886**	***−0.532***	**0.886**	0.774	0.245	0.542
**Nutty**	***−0.206***	0.831	0.606	0.401	***−0.049***	***−0.445***	0.674	***−0.262***	0.640	0.706	0.665	0.566	***−0.157***	***−0.580***	0.484
**Astringency**	***−0.387***	**0.921**	0.488	0.769	0.470	0.850	***−0.412***	0.576	***−0.254***	0.497	0.730	0.373	***−0.249***	***−0.471***	0.479
**Briny**	***−0.250***	0.478	***−0.043***	0.470	0.620	0.560	0.386	***−0.477***	0.530	0.513	***−0.133***	0.183	0.609	0.735	0.474
**Ripeness**	0.420	0.093	0.674	0.454	0.760	***−0.309***	0.690	0.559	0.618	***−0.106***	0.832	***−0.057***	0.444	0.373	0.473
**Buttery**	0.036	0.065	0.691	***−0.266***	0.600	0.405	0.751	0.537	0.407	***−0.353***	0.607	0.309	0.480	0.582	0.444
**Skin red**	**0.862**	0.356	0.551	**−0.063**	0.676	0.257	0.444	0.154	0.585	0.387	0.517	0.228	0.776	0.223	0.416
**Chewiness**	0.135	0.269	−0.045	0.485	0.235	0.568	0.646	0.476	0.311	0.414	0.046	0.677	0.406	0.811	0.410
**Vinegary**	**0.989**	0.255	0.798	**0.853**	0.392	0.079	0.422	-	**−0.277**	***−0.723***	**−0.511**	***−0.696***	**0.982**	0.594	0.392
**Oak barrel**	0.696	0.401	0.556	0.511	0.250	0.117	***−0.058***	0.367	0.546	0.118	0.640	0.334	***−0.426***	***−0.209***	0.351
**Bitterness**	***−0.145***	0.754	0.433	***−0.312***	0.590	0.533	0.735	0.127	0.158	0.740	0.245	0.053	***−0.039***	0.450	0.339
**Saltiness**	0.682	**0.850**	0.061	0.397	0.440	0.607	***−0.033***	0.792	0.531	0.060	***−0.312***	0.244	0.314	0.170	0.279
**Earthy soil**	0.703	-	***−0.843***	0.013	0.462	***−0.133***	0.478	0.544	0.328	0.239	***−0.283***	***−0.067***	***−0.392***	0.540	0.329
**Mouth coating**	0.770	0.770	***−0.558***	0.172	***−0.189***	0.283	***−0.143***	***−0.511***	0.406	**0.870**	0.434	0.817	***−0.393***	***−0.255***	0.228
**Natural fruity/floral**	***−0.514***	-	0.773	0.765	0.546	0.210	***−0.700***	0.685	***−0.482***	**0.952**	0.464	***−0.013***	0.055	***−0.798***	0.210
**Mushroom**	***−0.064***	0.473	0.023	0.834	0.386	0.408	0.286	0.152	0.076	0.343	***−0.070***	0.054	0.003	0.229	0.190
**Cheese smell**	0.237	0.050	0.258	***−0.058***	***−0.037***	0.379	***−0.241***	0.830	***−0.403***	0.846	0.478	***−0.652***	0.090	0.358	0.164
**Gassy smell**	0.695	0.537	-	***−0.099***	0.155	***−0.132***	-	0.138	***−0.037***	0.104	***−0.464***	0.192	0.341	0.180	0.146
**Alcohol**	***−0.294***	***−0.119***	0.526	0.805	***−0.192***	0.684	0.735	***−0.306***	0.389	0.011	***−0.292***	***−0.423***	0.798	0.260	0.135
**Soapy smell/medicinal**	0.134	***−0.387***	***−0.256***	**0.865**	0.279	0.795	**0.928**	***−0.370***	0.354	**0.865**	***−0.099***	***−0.056***	0.075	0.070	0.105
**Sourness**	0.605	***−0.037***	0.532	0.540	0.320	***−0.213***	0.123	***−0.149***	0.061	0.241	***−0.056***	***−0.414***	0.667	***−0.287***	0.092
**Rancid**	**0.932**	-	***−0.407***	0.694	0.038	0.038	***−0.108***	0.423	***−0.397***	0.568	0.589	***−0.132***	-	0.077	0.058
**Metallic**	0.466	***−0.117***	***−0.169***	0.307	0.700	**0.915**	***−0.601***	0.543	***−0.311***	***−0.061***	***−0.153***	0.776	0.087	***−0.527***	0.028
**Artificial fruity/floral**	0.597	0.579	0.310	0.359	0.441	***−0.337***	***−0.058***	0.103	***−0.217***	***−0.219***	***−0.137***	0.517	***−0.054***	***−0.054***	0.024
**Residual**	0.680	0.842	***−0.859***	0.068	***−0.500***	***−0.204***	***−0.731***	0.522	***−0.727***	0.760	***−0.680***	**0.876**	***−0.384***	***−0.234***	**−0.217**
**Median**	0.597	0.558	0.493	0.470	0.441	0.408	0.404	0.395	0.385	0.387	0.344	0.334	0.324	0.245	0.400

Notes: Significant agreement is indicated in bold while opposed behavior is shown in bold and italic.
